# Paclitaxel Induces Epidermal Molecular Changes and Produces Subclinical Alterations in the Skin of Gynecological Cancer Patients

**DOI:** 10.3390/cancers14051146

**Published:** 2022-02-23

**Authors:** Paula Montero, Martín Pérez-Leal, Jose Alejandro Pérez-Fidalgo, Celia Sanz, Cristina Estornut, Inés Roger, Javier Milara, Andrés Cervantes, Julio Cortijo

**Affiliations:** 1 Department of Pharmacology, Faculty of Medicine, University of Valencia, 46010 Valencia, Spain; celia.sanz@uv.es (C.S.); cristina.estornut@uv.es (C.E.); irola3@alumni.uv.es (I.R.); milara_jav@gva.es (J.M.); julio.cortijo@uv.es (J.C.); 2 Faculty of Health Sciences, Universidad Europea de Valencia, 46010 Valencia, Spain; martin.perez@universidadeuropea.es; 3 Department of Medical Oncology, Hospital Clínico Universitario de Valencia, 46010 Valencia, Spain; jopefi@uv.es; 4 Biomedical Research Networking Centre on Cancer (CIBERONC), Health Institute Carlos III, 28029 Madrid, Spain; 5 INCLIVA Biomedical Research Institute, 46010 Valencia, Spain; andres.cervantes@uv.es; 6 Health Sciences, Pre-Departmental Section of Medicine, Jaume I University of Castellón de la Plana, 12071 Castellón, Spain; 7 Biomedical Research Networking Centre on Respiratory Diseases (CIBERES), Health Institute Carlos III, 28029 Madrid, Spain; 8 Pharmacy Unit, University General Hospital Consortium, 46014 Valencia, Spain; 9 Research and Teaching Unit, University General Hospital Consortium, 46014 Valencia, Spain

**Keywords:** paclitaxel, skin alterations, epidermis, 3D epidermis model

## Abstract

**Simple Summary:**

Skin toxicity is one of paclitaxel’s adverse effects. However, its real impact on the skin could be underestimated as these alterations can also appear asymptomatic. We have observed that paclitaxel modifies gene and protein expression of skin markers in a 3D epidermis model, and impairs physical, physiological, and biomechanical properties of the skin in gynecologic cancer patients. These subclinical alterations might be avoided by using prophylactic measures during treatment to prevent possible future adverse reactions.

**Abstract:**

Background: Paclitaxel is a microtubule-stabilizing chemotherapeutic agent. Despite its widespread use, it damages healthy tissues such as skin. The goal of this study was to prove that the real impact of paclitaxel-induced skin toxicity could be underestimated because the adverse events might appear asymptomatic. Methods: Gynecological cancer patients were recruited. Skin parameters measurements were taken after three and six paclitaxel cycles. Measurements were conducted using specific probes which measure hydration, transepidermal water loss (TEWL), sebum, elasticity and firmness, erythema, roughness, smoothness, skin thickness, and desquamation levels. Further, a 3D epidermis model was incubated with paclitaxel to analyze gene and protein expression of aquaporin 3, collagen type 1, elastin, and fibronectin. Results: Paclitaxel induced alterations in the skin parameters with no visible clinical manifestations. Gynecological cancer patients under paclitaxel treatment had a decrease in hydration, TEWL, sebum, elasticity, and thickness of the skin, while erythema, roughness, and desquamation were increased. The molecular markers, related to hydration and the support of the skin layers, and analyzed in the 3D epidermis model, were decreased. Conclusions: Results suggest that paclitaxel modifies gene and protein expression of skin-related molecular markers, and impairs different physical, physiological, and biomechanical properties of the skin of cancer patients at a subclinical level.

## 1. Introduction

Taxanes are chemotherapeutic agents that produce antitumor activity by causing stabilization of microtubules, thereby inhibiting cell cycle progression [1]. Paclitaxel (PTX) is the prototype of the taxane family of antitumor compounds and binds to the β-tubulin subunit in the microtubule, leading to its stabilization and increasing microtubule polymerization [2]. This unique mechanism of action differentiates paclitaxel from other antimicrotubule agents such as vinca alkaloids or colchicine, which inhibit tubulin polymerization. The microtubules formed in the presence of paclitaxel are so stable that they cause cell death by disrupting the normal microtubule dynamics required for cell division and interphase processes [3]. The consequent arrest of the cell cycle has been considered as the cause of paclitaxel-induced cytotoxicity. However, the signaling pathways that lead to apoptosis are not well understood. Recent discoveries indicate that paclitaxel initiates apoptosis through multiple mechanisms [4].

In 1992, paclitaxel was approved by the US Food and Drug Administration (FDA) for the treatment of ovarian cancer. In 1996, a study of the Gynecologic Oncology Group (GOG) showed that paclitaxel-cisplatin was superior in terms of survival to the cyclophosphamide-cisplatin regimen as upfront therapy in stage III–IV ovarian cancer patients [5]. These results were confirmed by the European-Canadian Intergroup study [6]. These data justified the use of paclitaxel and platinum combination, and the treatment has become the standard of care in the first line. In the platinum-resistant setting, weekly paclitaxel has been considered one of the recommended regimens [7]. In endometrial and cervical cancer, paclitaxel in combination with platinum has also become part of the standard regimens in the first-line treatment. From this point, paclitaxel has been also used in the treatment of other cancers including colorectal and breast cancer, head and neck cancers, small-cell and non-small-cell lung cancers, and AIDS-related Kaposi Sarcoma [8].

Although taxanes are tolerable and manageable, their toxic profile includes a wide number of adverse events. Hematological, cardiologic, and neurologic toxicities are very common in taxane-containing regimens [9]. Neutropenia is described amongst the principal toxic effects of PTX and is dose-limiting [10]. Peripheral neuropathy, another dose-dependent side effect, is found in 60–70% of chemotherapy patients and is characterized by sensory symptoms, such as numbness and paresthesia [11]. One of the most frequent adverse events is taxane-induced dermatologic toxicity, which has been reported in up to 89% of patients [12]. The spectrum of cutaneous reactions to paclitaxel includes alopecia, hypersensitivity reactions such as erythema and urticaria, nail changes, and radiation recall dermatitis. Less common effects such as acral erythema, erythema multiforme, pustular dermatitis, and scleroderma-like changes have also been described [12,13]. Generally, the adverse effects on the skin are mild to moderate in severity and self-limiting. Consequently, they are usually dose-dependent and sometimes require dose reductions, interruptions, or termination of the taxane chemotherapy [14].

There are scarce data regarding the mechanisms that lead to these toxic effects and most remain not understood. Moreover, the real impact of the taxane-induced skin toxicity could be underestimated as the skin adverse events are usually under-reported or paucisymptomatic [14]. The available data in vivo are limited to case reports and oncology studies, and usually describe events happening to symptomatic patients. There is no information regarding the impact of taxanes on the skin in patients without cutaneous symptoms. However, a direct cytotoxic effect of chemotherapy on basal keratinocytes has been proposed; histology from skin biopsies of PTX treated patients have shown alterations in keratinocytes when some cutaneous events occur [15,16,17]. Further, studies in vitro have described that paclitaxel induces a cytotoxic response in transformed HaCat keratinocytes [18] and produces epithelial damage in zebrafish models [19,20]. Further, our previous results showed that paclitaxel impacts on the expression of proteins related to angiogenesis, elasticity, inflammation, and senescence in human keratinocytes [21].

Of note, studies on undifferentiated keratinocyte monolayer cultures can lack some of the physiological functions of the stratified keratinocyte epithelium and could misinterpret the results obtained in preclinical studies. Thereby, various three-dimensional (3D) skin equivalents reproducing in vivo conditions have been developed for pharmacologic and toxicologic in vitro testing as an alternative to animal models [22,23]. One of these models is characterized by the growth of keratinocytes on a feeder layer of lethally irradiated 3T3 fibroblasts. The feeder layer supports and maintains keratinocyte colony growth and stratification [24,25], producing a 3D model that is compatible with autologous and allogenic transplantation [26,27].

In this study, we aimed to overcome the lack of investigation regarding subclinical alterations induced by PTX. Therefore, we analyzed PTX-induced subclinical skin alterations by measuring different biomechanical properties of the skin in oncologic patients. Secondly, we reconstructed a 3D epidermis cell model to mimic a healthy epidermis and evaluate the effects of paclitaxel in some of the molecular markers associated with skin homeostasis. The results obtained will help understand asymptomatic skin alterations to prevent possible future skin adverse effects.

## 2. Materials and Methods

### 2.1. Patients

This project was approved by the Research Ethics Committee of Valencia University Clinical Hospital and further authorized by the Valencian Regional Ministry of Health. Informed consent was obtained from each participant before starting the study. Twenty cancer patients and 20 healthy volunteers were recruited from the oncology service at Valencia University Clinical Hospital. The patients’ clinical features can be found in Table 1. Inclusion criteria for the control group comprised being Caucasian females aged 40–70 years old. Exclusion criteria included having an acute illness, skin pathologies, being pregnant, or breastfeeding. The inclusion criteria for cancer patients included: (1) being over 18 years old; (2) having a clinical diagnosis of gynecological cancer (ovarian, cervix or endometrium cancer at any stage of the International Federation of Gynecology and Obstetrics (FIGO) classification); (3) having indication for treatment with taxanes; (4) being treated for the first time or in relapse; (5) having adequate kidney, liver, and hematological functions before treatment; or (6) having PTX prescription on a 3 week schedule in combination or PTX in a weekly schedule either in monotherapy or in combination. Exclusion criteria comprised (1) having known chronic or rheumatologic skin disease; (2) being under corticosteroid treatment 2 weeks before admission to the study; (3) acute illness; (4) being pregnant or breastfeeding; or (5) having visible skin adverse effects. During the study, patients were not allowed to use cosmetic treatments.

### 2.2. Skin Parameters Measurements

Skin parameter measurements were performed in three visits: before treatment (T1), during treatment after 3 chemotherapy cycles (T2), and at the end of treatment after 6 chemotherapy cycles (T3). The measurements were taken using specific probes following the measurement guidelines [28,29,30]. All probes were purchased from Courage–Khazaka Electronic (Cologne, Germany).

Corneometer CM 825^®^ (Courage-Khazaka Electronic, Cologne, Germany) was used to obtain cheekbone and forearm hydration values. This probe measures the electrical capacity of the stratum corneum, based on the linear dependency of the electrical property of the epidermis to its hydration. The results are displayed in arbitrary units [28].

Tewameter TM 300^®^ (Courage-Khazaka Electronic, Cologne, Germany) was used to provide cheekbone and forearm values of transepidermal water loss (TEWL). The probe measures the vapor pressure and calculates TEWL from the difference between two measurement points using Fick’s law of diffusion. It displays the results in grams per hour per square meter (g/hm^2^) [31].

Sebumeter SM 815^®^ (Courage-Khazaka Electronic, Cologne, Germany) was used to obtain the forehead sebum value. The probe determines the translucency of a special tape, which becomes transparent after contact with sebum on the skin surface and displays the sebum values in µg per square centimeter (µg/cm^2^) [32].

Mexameter MX 18^®^ (Courage-Khazaka Electronic, Cologne, Germany) was used to determine the forearm erythema based on tissue’s narrow wavelength light absorption. Results are displayed as erythema index in arbitrary units [29].

Skin elasticity and firmness were assessed in the cheekbone with Cutometer^®^ MPA 580. This probe suctions skin and gives a series of R values. The ones that provide more information about elasticity and firmness are R0, R2, R5, and R7. R0 expresses the maximum width of skin and is given in mm. R2 represents the ratio between the maximum width of the skin and its ability to return to its original state after suction (Ua/Uf). R5 represents the ratio between the elasticity of the suction phase and the elasticity of the relaxation phase (Ur/Ue). R7 represents the elastic recovery ratio (Ur/Uf). The closer R2, R5, and R7 are to 1, the greater the elasticity.

Skin smoothness and roughness were assayed in the cheekbone by Visioscan^®^ (Courage-Khazaka Electronic, Cologne, Germany). The device takes grayscale photographs to study the epidermis surface and analyzes the obtained images, where white and bright gray are associated with a bad condition of the skin. The software uses the images to determine the skin topography parameters SELS (surface evaluation of the living skin) [33]. The given parameters are smoothness (Sesm) and roughness (Ser) and are expressed in arbitrary units. Low values of Sesm inform about smoother skin, while high values of Ser imply rougher skin [34].

Visioscan^®^ was also combined with the Corneofix^®^ (Courage-Khazaka Electronic, Cologne, Germany) technique to obtain the skin desquamation index on the forehead. Corneofix^®^ is a tape that adheres to the skin and collects corneocytes. Then, the tape is placed on the Visioscan^®^ probe which takes an image and processes it with a determined color grading, depending on the desquamation level. The color scale ranges from cool to warm colors. The software also analyzes the number, size, and thickness of the attached corneocytes to the Corneofix^®^ tape and gives the desquamation percentage.

Finally, Ultrascan^®^ UC22(Courage-Khazaka Electronic, Cologne, Germany) was used to analyze the thickness of the different layers of the skin on the forearm. The probe takes an ultrasound image of the skin.

All measurements were taken under controlled conditions. Temperature was maintained at 22 ± 2 °C and relative humidity between 40% and 60%. Measurements were taken after patients remained in a 30 min acclimatization period in the same atmospheric conditions.

### 2.3. Epidermis Cell Model Reconstruction

Three-dimensional epidermis cell models were reconstructed using the BALB/3T3 feeder-layer technique adapted from Mak et al. [35] and Arnette et al. [24]. In brief, 10^6^ BALB/3T3 fibroblasts (Lonza, Basel, Switzerland) were seeded on collagen-coated Millicell inserts (Millicell-CM 12 mm, transparent Biophore Membrane; Millipore Corp., Bedford, MA, USA) and placed into 6-well plates (Corning Incorporated, Corning, NY, USA). Fibroblasts were cultured for 2 days in 1 mL Dulbecco’s Modified Eagle Medium (DMEM, high glucose; Gibco, Waltham, MA, USA) supplemented with 10% fetal calf serum (FCS, Gibco, Waltham, MA, USA) and added to the apical and dorsal side of the insert. When fibroblasts reached 60–70% confluence, the monolayer was irradiated with UV light at 0.048 mW for 1 h with UVACUBE 400 (Honle UV Technology, Gräfelfing, Germany) to establish the feeder layer. Then, primary adult epidermal keratinocytes (192627, Lonza, Basel, Switzerland) were seeded at a density of 0.5 × 10^6^ cells/cm^2^. Cultures were grown at 37 °C and 95% air/5% CO_2_ until approximately 60% confluency and then were switched to Keratinocyte Growth Medium (KGM-Gold™, Lonza, Basel, Switzerland) supplemented with KGM-Gold SingleQuot Kit (Lonza) until confluent. Confluent cultures were raised to the air–liquid interface and cultured for 21 days until epidermal stratification was achieved. To validate the stratification, histological analysis was performed after 21 days. The reconstructed epidermis tissues were fixed with 10% formalin solution, dehydrated, and embedded in paraffin. Six-micrometer-thick sections were cut and stained with hematoxylin–eosin. Random photographs were taken of each sample with a Leica DM6000B microscope (Leica Biosystems, Wetzlar, Germany).

### 2.4. Real Time RT-qPCR

The 3D epidermal cell models were incubated for 24 h with PTX within the clinically achievable plasma concentrations of 0.3, 3, and 30 µM [36,37,38]. After incubation, total RNA was extracted using TRIzol^®^ Reagent (Invitrogen, Thermo Fisher Scientific, Waltham, MA, USA) following the manufacturer’s instructions. Reverse transcription was performed in 500 ng of total RNA with a TaqMan reverse transcription reagents kit (Applied Biosystems, Thermo Fisher Scientific, Waltham, MA, USA). cDNA was amplified with specific primers and probes predesigned by Applied Biosystems for aquaporin 3 (AQP3) (Hs00185020_m1), collagen type 1 (COL1) (Hs00164004_m1), elastin (ELN) (Hs00355783_m1), and fibronectin (FN1) (Hs01549976_m1) in a QuantStudio™ 5 Real-Time PCR System, using universal master mix (Applied Biosystems, Thermo Fisher Scientific, Waltham, WA, USA). Expression of the target gene was expressed as the fold increase or decrease relative to the expression of β-actin (Hs01060665_g1) as an endogenous control. The mean value of the replicates for each sample was calculated and expressed as the cycle threshold (Ct). Gene expression level was calculated as the difference (ΔCt) between the Ct value of the target gene and the Ct value of β-actin. The fold changes in the target gene mRNA levels were designated 2^−ΔCt^.

### 2.5. Western Blotting Analysis

The 3D epidermal cell models were incubated for 24 h with different PTX concentrations (0.3, 3, and 30 µM). After incubation, protein extraction was performed incubating samples with lysis buffer (1M HEPES, 4 M NaCl, 0.5 M EDTA, 0.1 M EGTA) supplemented with a protease inhibitory cocktail complete™ and phenyl-methyl-sulfonyl fluoride (PMSF) (Roche Diagnostics, Indianapolis, IN, USA). Total protein concentration was quantified using the BCA Protein Assay Kit (Thermo Fisher Scientific, Waltham, MA, USA). Protein electrophoresis was performed to separate proteins according to their molecular weight. Twelve micrograms of denatured proteins along with Rainbow ™ molecular weight marker (Sigma-Aldrich, Saint Louis, MO, USA) were loaded into Mini-PROTEAN^®^ polyacrylamide gels TGX™ (Bio-Rad, Herts, UK) by application of 100 V during 1 h. Proteins were transferred from the gel to a nitrocellulose membrane Trans-Blot^®^ Turbo™ Transfer Pack, using the Trans-Blot^®^ Turbo™ Transfer System (Bio-Rad Laboratories, Herts, UK). Then, membranes were incubated with 5% bovine serum albumin (BSA) for 2 h and labeled overnight at 4 °C, with various primary antibodies. The secondary antibody was incubated for 1 h at room temperature. The primary antibodies used were the following: AQP3 (ab125219, Abcam, Cambridge, UK); COL1A (PA5-95137, Thermo Fisher Scientific, Waltham, MA, USA); FN1 (PA5-29578, Thermo Fisher Scientific, Waltham, MA, USA); and ELN (ab23747, Abcam, Cambridge, UK). To normalize results, the β-actin antibody (A1978, Sigma-Aldrich, Saint Louis, MO, USA) was used as housekeeping control. Signal visualization of proteins was carried out by incubating the membranes with chemiluminescence reagents (ECL Plus, Amersham GE Healthcare, Buckinghamshire, UK). Densitometry of films was performed using Image J 1.42q software. Results of target protein expression are expressed as the percentage of the densitometry of the endogenous controls β-actin.

### 2.6. Statistical Analyses

Results from cellular in vitro experiments were expressed as the mean ± standard error (SE) of *n* experiments; *p* < 0.05 was considered statistically significant. Normal distribution for each data set was confirmed by the Kolmogorov–Smirnov test. Statistical analysis was carried out by multiple comparisons with analysis of variance (ANOVA) followed by Bonferroni post hoc test. Results from the human in vivo experiments were expressed as the mean ± standard error (SE) of *n* experiments; *p* < 0.05 was considered statistically significant. When the comparisons concerned only 2 groups (healthy vs. PTX T1), statistical analysis was carried out by unpaired *t*-test. Multiple comparisons were analyzed by ANOVA followed by the Bonferroni post hoc test.

## 3. Results

### 3.1. Paclitaxel Affects the Hydration Levels in the Skin

**Figure 1:**
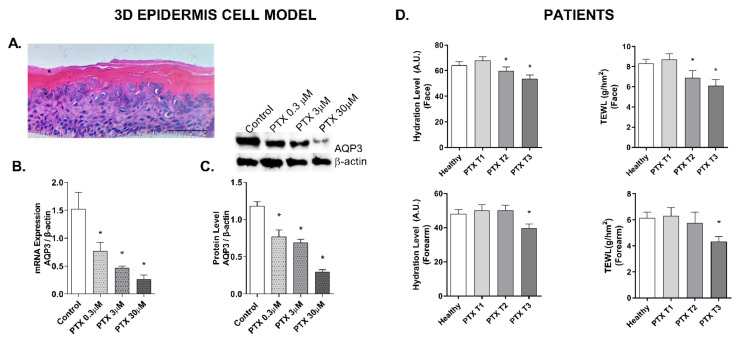
Paclitaxel induces a dose-dependent loss of hydration levels in oncologic patients and reduces the expression of the hydration marker AQP3 in the 3D epidermal cell model. (**A**) Paraffin section from the 3D epidermis model stained with hematoxylin and eosin. Scale bar 100 µm. (**B**) Three-dimensional epidermal cell model tissues were incubated for 24 h with increasing paclitaxel (PTX) concentrations. Aquaporin (AQP3) mRNA levels were measured by real-time PCR. Data are expressed as 2^−ΔCt^. (**C**) Three-dimensional epidermal cell model tissues were incubated for 24 h with increasing paclitaxel (PTX) concentrations. AQP3 protein levels were analyzed by Western blotting. Quantification was performed by densitometry and normalized to β-actin. Results are expressed as the mean ± standard deviation of two independent experiments (*n* = 3); * *p* < 0.05 vs. control. Uncropped Western Blots can be found at Supplementary File. (**D**) Hydration levels were measured in 20 oncologic patients before (T1), during (T2), and after (T3) treatment with PTX, and in 20 healthy subjects as a control group. Measurements were conducted with Corneometer CM 825^®^ in the cheekbone and forearm. Transepidermal water loss (TEWL) levels were measured in 20 oncologic patients before (T1), during (T2), and after treatment (T3) with PTX, and in 20 healthy subjects as a control group. Measurements were conducted with Tewameter TM 300^®^ in the cheekbone and forearm. Results are expressed as the mean ± standard deviation of at least 3 measurements each time (*n* = 20); * *p* < 0.05 vs. T1.

The stratification of the 3D epidermis cell model was confirmed by the hematoxylin–eosin staining. As shown in Figure 1A, keratinocytes were distributed into the principal epidermis layers: basal, spinous, and granular, and its terminal differentiation resulted in the presence of the stratum corneum, analogously to the epidermal in vivo structure of healthy skin.

The effects of PTX treatment in the hydration molecular marker aquaporin (AQP3) in the 3D skin model were examined. Incubation with PTX for 24 h induced a dose-dependent decrease in gene expression that was statistically significant (Figure 1B). Further, incubation of the 3D skin model with PTX for 24 h induced a similar significant decrease in AQP3 protein expression in all doses (Figure 1C). Hydration was also measured with Corneometer^®^ on the cheekbone and forearm of the oncologic patients and to the control group (Figure 1D). No statistically significant differences were observed between the values of the control subjects and the values of cancer patients before treatment (T1). However, after three (T2) and six (T3) PTX cycles, both areas of skin manifested reduced hydration values. At the T3 timepoint, the variation percentages were −18.73% ± 5.42 and −16.38% ± 5.67 in the cheekbone and forearm, respectively. TEWL was also examined in the cheekbone and forearm with Tewameter^®^ (Figure 1D). No statistically significant differences were observed in TEWL between the control group and the cancer patients before treatment (T1). Chemotherapeutic treatment with PTX decreased the TEWL value in the second (T2) and third (T3) visits. The differences were statistically significant in the forearm area at the T3 timepoint, and in the cheek area at T2 and T3 timepoints (Figure 1B). Medium TEWL percentage variations at T3 were −26.67% ± 8.63 in the cheekbone and −18.56% ± 9.49 in the forearm.

### 3.2. Paclitaxel Induces a Decrease in Elasticity and Firmness of the Skin

**Figure 2:**
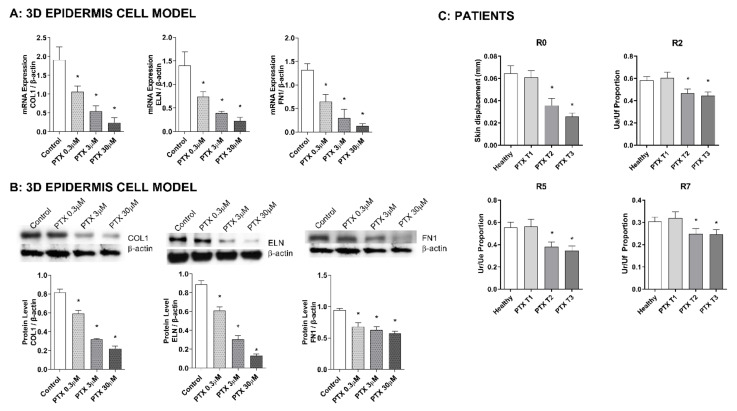
Paclitaxel impairs skin elasticity and firmness in oncologic patients and reduces the expression of elasticity and firmness molecular markers in a 3D epidermis model. (**A**) The 3D epidermis models were incubated for 24 h with increasing paclitaxel (PTX) concentrations. Collagen type 1 (COL1), elastin (ELN), and fibronectin (FN1) mRNA levels were measured by real-time PCR. Data are expressed as 2^−ΔCt^. (**B**) The 3D epidermis models were incubated for 24 h with increasing PTX concentrations. COL1, ELN, and FN1 protein levels were analyzed by Western blotting. Quantification was performed by densitometry and normalized to β-actin. Results are expressed as the mean ± standard deviation of two independent experiments (*n* = 3); * *p* < 0.05 vs. control. Uncropped Western Blots can be found at Supplementary File. (**C**) The elasticity and firmness parameters R9, R2, R5, and R7 were measured in 20 oncologic patients before (T1), during (T2), and after (T3) treatment with PTX, and in 20 healthy subjects as a control group. Measurements were conducted with Cutometer^®^ MPA 580 probe in the cheekbone. Results are expressed as the mean ± standard deviation of at least 3 measurements each time (*n* = 20); * *p* < 0.05 vs. T1.

Incubation of the 3D skin model with PTX for 24 h induced a decrease in the gene expression of the three analyzed skin elasticity and firmness markers: COL1, ELN, and FN1. The mRNA downregulation was dose-dependent (Figure 2A). In the same way, treatment with PTX for 24 h induced the same response at the protein level, decreasing COL1, ELN, and FN1 protein expression in all doses (Figure 2B).

The effect of PTX on the elasticity and firmness of the skin was also evaluated in oncologic patients using Cutometer^®^ (Figure 2C). In all parameters analyzed no differences were observed between the healthy volunteers and the oncologic patients before treatment (T1). However, treatment with PTX induced significant reductions in all values at T2 and T3 timepoints. The mean variations at T3 were R0: −47.21% ± 8.33, R2: −19.69% ± 6.10, R5: −33.06% ± 5.92, and R7: −19.40% ± 7.09.

### 3.3. Paclitaxel Affects Sebum and Erythema Levels in the Skin

**Figure 3:**
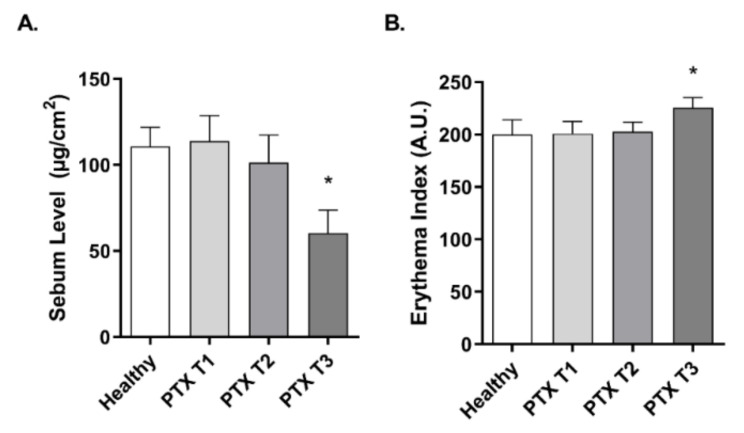
Paclitaxel reduces sebum levels and increases erythema in oncologic patients. (**A**) Sebum levels were measured in 20 oncologic patients before (T1), during (T2), and after (T3) treatment with PTX, and in 20 healthy subjects as a control group. Sebumeter SM 815^®^ was used to obtain the forehead sebum value. (**B**) The erythema index was measured in 20 oncologic patients before (T1), during (T2), and after treatment (T3) with PTX, and in 20 healthy subjects as a control group. Mexameter MX 18^®^ was used to determine the forearm erythema. Results are expressed as the mean ± standard deviation of at least 3 measurements each time (*n* = 20); * *p* < 0.05 vs. T1.

The effects of PTX on skin lipids were evaluated by measuring the skin sebum production with Sebumeter^®^ (Figure 3A), and its effects on skin redness were evaluated by measuring the erythema value obtained with Mexameter^®^ (Figure 3B). In both parameters, no differences were encountered between the healthy group and the oncologic group previously to treatment (T1). After three PTX cycles (T2), the sebum levels were slightly reduced but not significant, while the erythema values remained constant. After six PTX cycles (T3), sebum levels were reduced significantly with a variation percentage of −45.29% ± 8.23 and the erythema value increased with a mean variation of 13.96% ± 4.11.

### 3.4. Paclitaxel Impairs Smoothness and Increases Roughness of Skin in Oncologic Patients

**Figure 4:**
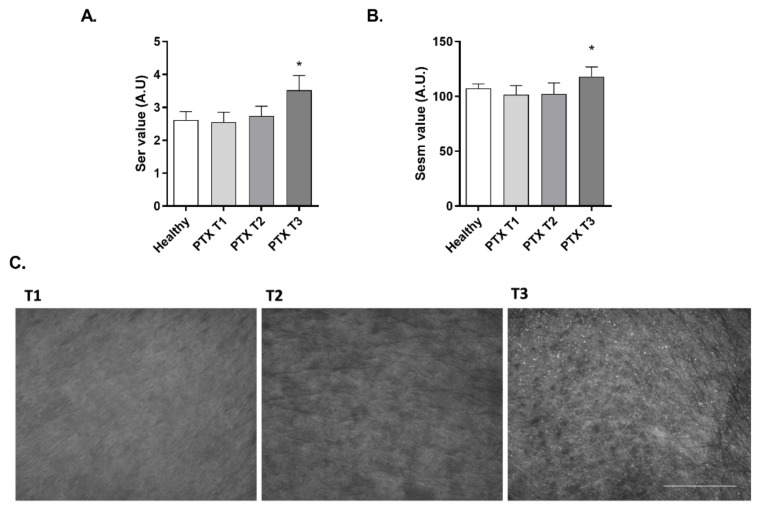
Paclitaxel induces an increase of skin roughness and reduces its smoothness in cancer patients. (**A**,**B**) Skin roughness and smoothness were measured in 20 oncologic patients before (T1), during (T2), and after (T3) treatment with paclitaxel (PTX), and in 20 healthy subjects as a control group. Measurements were conducted in the cheekbone by Visioscan^®^ VC 98 probe, obtaining the Ser and Sesm values calculated by the software from each grayscale photograph. Results are expressed as the mean ± standard deviation of at least 3 measurements each time (*n* = 20); * *p* < 0.05 vs. T1. (**C**) Representative images of the skin topography obtained with Visioscan^®^ during PTX treatment at time points T1, T2, and T3. White and bright gray on the images are associated with a bad condition of the skin. Scale bar 1 mm.

The smoothness (Sesm) and roughness (Ser) parameters were also evaluated on the skin of oncologic patients with the Visioscan^®^ equipment. As seen on the representative images in Figure 4C, treatment with PTX worsened the aspect of the skin surface at T2 and T3 timepoints. The Visiosca^®^ software analyzed each image and displayed the Ser (Figure 4A) and Sesm (Figure 4B) parameters. Ser is directly proportional to roughness and Sesm is inversely proportional to smoothness. No differences were observed between the Ser and Sesm values of healthy volunteers and oncological patients before treatment (T1). However, chemotherapeutic treatment with PTX produced an increase in Ser and Sesm values, after six cycles of PTX (T3) (Figure 4A,B), which represent a loss in skin smoothness and an increased roughness. The mean Ser variation at T3 was 49.02% ± 17.94 and the mean Sesm variation was 20.54% ± 5.80.

### 3.5. Paclitaxel Increases the Skin Desquamation Levels in Oncologic Patients

**Figure 5:**
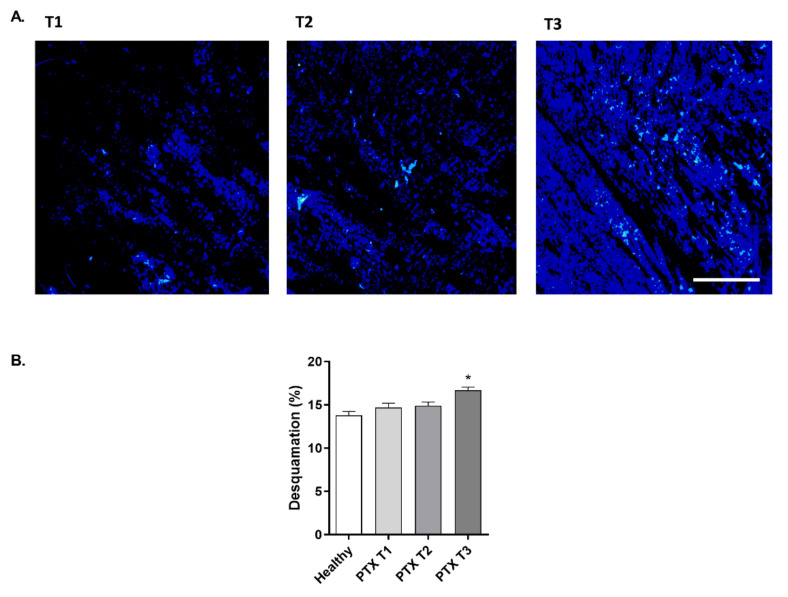
Paclitaxel increases the skin desquamation levels in oncologic patients. (**A**) Representative Corneofix^®^ tape images obtained before (T1), during (T2), and after (T3) treatment with paclitaxel. Images were obtained with the Visioscan^®^ probe. The blue color on the image represents higher desquamation levels. Scale bar 1 mm. (**B**) Skin desquamation percentage was measured in 20 oncologic patients after PTX treatment at T1, T2, and T3 timepoints and in 20 healthy subjects as a control group. Measurements were conducted on the forehead with the combination of the Corneofix^®^ tape and the Visioscan^®^ probe. Results are expressed as the mean ± standard deviation of at least 3 measurements each time (*n* = 20); * *p* < 0.05 vs. T1.

The effect of PTX on skin desquamation was evaluated by combining the Corneofix^®^ tape sheets with the Visioscan^®^ equipment. Visioscan^®^ software displays a color-graded image of the Corneofix^®^ tape with the attached corneocytes. As shown in the representative images in Figure 5A, higher blue staining at T2 and T3 represent higher desquamation levels. The analyzed desquamation percentage is shown in Figure 5B. While no differences were observed between the percentage of desquamation of healthy volunteers and cancer patients at T1, treatment with PTX increased skin peeling, with statistically significant differences after six PTX cycles (T3). The mean variation at T3 compared to T1 was 14.94% ± 3.17.

### 3.6. Paclitaxel Reduces Skin Thickness in Oncologic Patients

**Figure 6:**
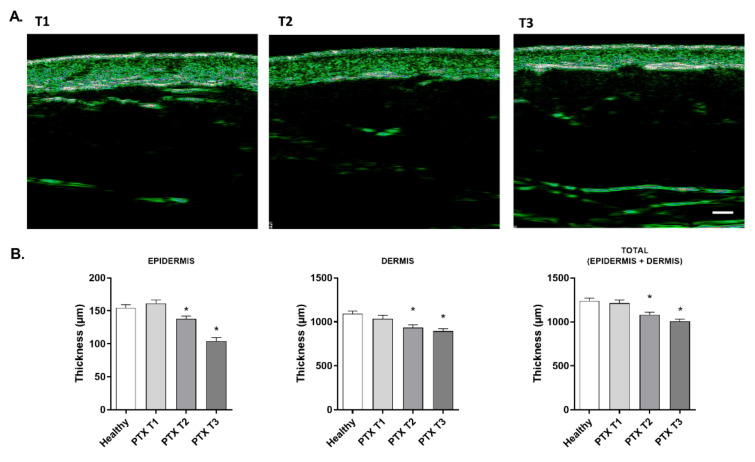
Paclitaxel reduces skin thickness in oncologic patients. (**A**) Representative ultrasound images of the skin thickness obtained during treatment with PTX in 20 oncologic patients before (T1), during (T2), and after (T3) treatment. Images were obtained with Ultrascan^®^ UC22. Scale bar 1 mm. (**B**) Epidermis, dermis, and total skin thickness measured in 20 oncologic patients under PTX treatment at timepoints T1, T2, and T3, and in 20 healthy subjects as a control group. Measurements were conducted on the forearm with Ultrascan^®^ UC22. Results are expressed as the mean ± standard deviation of at least 3 measurements each time (*n* = 20); * *p* < 0.05 vs. T1.

The ultrasound technique of Ultrascan^®^ UC22 was used to analyze the thickness of the different layers of the skin in oncologic patients. Epidermis, dermis, and the total thickness of the skin were similar in the healthy group and patients before treatment. After three and six PTX cycles, all layers of skin showed a decrease in thickness, which can be noted in the representative images in Figure 6A. The mean thickness variations in comparison to T1 were −34.37% ± 3.50 in epidermis, −12.75% ± 2.05 in the dermis, and −16.46% ± 1.58 for total skin thickness (Figure 6B).

## 4. Discussion

Paclitaxel is an antineoplastic drug widely used in cancer treatment that has been shown to produce a multitude of skin adverse effects [39]. However, the subclinical alterations caused by PTX on the skin have not previously been described. In this study, we investigated these events in oncologic patients under PTX treatment by measuring hydration, TEWL, sebum, elasticity, erythema, roughness, desquamation, and thickness of the skin. The study was carried out without the development of skin reactions. The use of the Courage–Khazaka Electronic probes allowed us to measure the different skin parameters without the need of performing histological analysis of skin biopsies. To support these findings with in vitro experiments, we used a 3D epidermis model of keratinocytes grown on a feeder layer and exposed to the air–liquid interface. Hematoxylin–eosin staining demonstrated the development of a fully differentiated epidermis. The 3D epidermis model was used as a mimicker of a healthy epidermis to evaluate molecular modulation induced after treatment with the clinically achievable plasma concentrations of PTX.

In this study, oncologic patients did not report visible cutaneous symptoms. The values of the skin properties in oncologic patients before treatment (T1) were compared to the values of a healthy control group. In all cases, the parameters were similar and no statistically significant differences were encountered between both groups. This shows that before treatment with PTX, all patients had skin parameters within the normal biological values, representative of the general population. Additionally, data obtained from healthy volunteers and cancer patients in their first visit were within the reference values, as described by the literature [29,30,40,41]. 

Firstly, we analyzed the hydration levels and TEWL. In the epidermis cell model, treatment with PTX reduced the gene and protein expression of the marker Aquaporin (AQP3). AQP3 is the most abundant skin aquaglyceroporin and is responsible for transporting water and glycerol in the epidermis. Therefore, AQP3 is a key protein in the maintenance of epidermal hydration and differentiation of keratinocytes [42,43,44]. Only one case report has related PTX and aquaporins previously: A patient under PTX treatment developed a cystoid macular edema induced by the functional failure of aquaporin mediated water transport [45]. This finding, in line with our results, suggests that PTX induces the modulation of aquaporins. In addition, aquaporins are reported to play a major role in angiogenesis, cell proliferation, apoptosis, and cell migration [46]. Thereby, PTX could be impairing other cellular processes through the modulation of AQP3. To see if these molecular changes could directly affect the skin in patients under PTX treatment, we measured hydration levels and transepidermal water loss (TEWL). In agreement with the in vitro results, patients showed reduced face hydration levels after three and six PTX cycles. Hydration values on the forearm were significantly reduced after six PTX cycles. This difference can be explained by the fact that the face is a photoexposed area, and the damage produced by sunlight on skin cells might enhance the dehydration induced by PTX [47,48]. Skin dehydration in oncologic patients can lead to the reduction of the skin water content. Indeed, patients showed a decrease in TEWL in comparison to the values obtained before treatment. TEWL is an indicator of the ability of the epidermis to hold water and is a good marker of the functionality of the skin barrier [30]. In patients with taxane-related xerosis, TEWL appears increased [14]. We suggest that these differences might be explained by the fact that patients with xerosis have a developed skin adverse effect, while patients in this study did not show any symptomatic alterations. The effects of PTX in this study represent the early modifications that could lead to the development of later adverse effects. Furthermore, because lipids act as a barrier against water loss [49], we also analyzed the sebum levels in oncologic patients. Sebum levels showed a progressive decrease with the progression of PTX treatment, which represents the loss of its skin protection layer. Overall, we can state that PTX proved to impair skin moisturization in oncologic patients and compromised the skin barrier function.

The epidermis cell model was also incubated with PTX to analyze collagen 1 (COL1), elastin (ELN), and fibronectin (FN1) expression. These proteins play a key role in maintaining the elasticity, firmness, and support of the skin layers [50,51,52]. Gene and protein expression of these markers was significantly reduced after PTX treatment on the 3D epidermis model. In agreement with these results, a study on tenon fibroblasts monolayers showed that both collagen and fibronectin were markedly downregulated in the culture medium [53,54]. There is also in vivo evidence that showed alterations in collagen in a skin biopsy from a sclerodermatous area of a patient under taxane treatment [55]. These results are directly related to those obtained in our in vivo study on cancer patients treated with PTX. Oncologic patients had decreased skin elasticity and firmness as shown by the lowered R parameters, which represent the state of the biomechanical elastic properties of the skin [56,57,58]. These results suggest that PTX-induced decrease in the skin elasticity and firmness might be mediated by its capacity to modulate molecular markers such as COL1, ELN, and FN1, which maintain the structure of the skin layers.

Erythema is a common adverse effect seen on patients under PTX treatment [13,59]. In this study, patients did not develop clinically visible erythema. However, we wanted to analyze the possible subclinical manifestations. Colorimetric examinations with Mexameter^®^ showed that the erythema index had a slight increase after six PTX cycles. It is widely described that treatment with taxanes induces erythema at different areas of skin and in different grades of severity [14,60,61]. However, this is the first study that reported erythemal changes on the skin under PTX treatment before the development of clinically visible erythematous skin reactions. 

As it was evident that PTX induced changes in the biomechanical properties of skin, roughness and softness were also examined. PTX induced an increase in skin roughness and a decrease in smoothness. Connected to these events, patients showed an increased desquamation percentage. This implies a decrease in the barrier function capacity of the skin after PTX treatment. Similar to the other parameters analyzed, the research data available comes from case reports of patients suffering from PTX-induced toxicities with clinical manifestations. In this case, desquamation had been previously described in patients under PTX treatment, in the palms and soles, necessitating treatment interruption [62] and associated with rash reactions [63]. Regarding the thickness of the skin, PTX induced the reduction of the dermis, epidermis, and total skin thickness. These results were expected, bearing in mind that PTX induced the reduction of the structural proteins COL1, FN1, and ELN, which causes an impairment of the skin’s architecture, and therefore changes in its thickness. This effect of PTX has not been described previously. However, there is a case report describing acanthotic epidermis in a PTX-induced cutaneous eruption [16], which indicates the potential of PTX to induce changes in the structure of the skin.

There are a few study limitations to be mentioned. Firstly, the heterogeneity of the sample—there are patients with localized disease and patients in different situations of advanced disease. Therefore, the chemotherapy administration schemes differ between patients. However, the heterogeneity of the sample should not have a significant impact since it includes only gynecological tumors with similar treatments. Secondly, considering the treatments used, the drug with the greatest impact at the cutaneous level was PTX. We acknowledge that carboplatin causes skin adverse effects but they are usually associated with hypersensitivity reactions [64]. As the number of patients was limited, and the purpose of the study was to analyze asymptomatic skin alterations, it was considered that the other drugs used in combination with PTX will not significantly bias our data. Finally, as not all patients were exposed to taxanes for the first time, a healthy population was included as a control group to prove that the baseline results of cancer patients before treatment were not statistically different from the healthy volunteers. This comparison served to prove that, in the patients included in the study, accumulated doses of paclitaxel did not affect the severity of the alterations.

The results of this study show that, although PTX did not cause severe skin adverse reactions, it impaired its physical, physiological, and biomechanical properties with no clinical manifestations. Treatment with PTX induced skin dehydration, a decrease in elasticity, thickness, and sebum levels, and an increase in skin desquamation and erythema. These changes were related to the modulation of gene and protein expression induced by PTX in an epidermis cell model that mimics a healthy epidermis. These results indicate that PTX can alter the skin structure, and impair its barrier function, inducing cutaneous changes that do not become symptomatic. Previous studies have described PTX-induced epithelial damage in zebrafish models [19,20] and some case reports show histologic changes caused by PTX on the skin [16,17]. However, to our knowledge, this is the first study that analyzes the subclinical alterations caused by PTX and it might explain the prediction of later severe cutaneous adverse events. Skin symptoms in patients cause physical pain and discomfort and psychological distress. In severe cases, skin toxicities can cause treatment delays and even discontinuation, which affects clinical outcomes [65]. This highlights the need for the early management of these alterations. There is a relative lack of evidence for effective management of taxane skin toxicities [66]. Some studies recommend using a scalp cooling system to reduce alopecia, frozen gloves to prevent nail and cutaneous hand changes [12], and nail solution to prevent chemotherapy-induced nail toxicity [67]. Additionally, a preliminary study in patients undergoing chemotherapy with taxanes demonstrated that resveratrol, lycopene, vitamin C, and anthocyanins (Ixor^®^) had a protective role against skin reactions [68]. However, there is still a lack of studies that propose prophylactic measures to prevent skin alterations in patients under paclitaxel treatment.

In conclusion, the results provided by this work suggest the need for prophylactic measures that improve the patient’s quality of life as well to ensure adherence to treatment. Although more studies addressing this matter are needed, early introduction of effective countermeasures including daily skincare (skin cleansing, moisturization, and irritation prevention) would help in the prevention of future PTX-induced skin toxicities.

## 5. Conclusions

This work has shown that paclitaxel impairs different physical, physiological, and biomechanical properties of the skin. To our knowledge, this is the first study that has concluded that gynecological cancer patients under paclitaxel treatment show subclinical skin alterations. These subclinical alterations include the decrease of hydration, TEWL, sebum, elasticity, and thickness of the skin, together with an increase in erythema, roughness, and desquamation. Further, this study showed the PTX-induced modulation of molecular markers related to hydration and the support of the skin layers in a 3D epidermis model. These altogether highlight the lack of management measures to prevent skin alterations in patients under taxane treatment. The use of prophylactic measures at the early stages of treatment could be useful to avoid these subclinical alterations and prevent future severe reactions.

## Figures and Tables

**Table 1:** Patients’ clinical features.

Nº	Age	Location	Tumor Subtype	Grade	FIGO Classification	Strategy	Treatment	Previous Taxane
1	63	Ovary	Serous carcinoma	High	III	Pt-sensitive relapse	Paclitaxel + Carboplatin + Bevacizumab	yes
2	60	Endometrium	Carcinosarcoma	High	IV	2nd line	Paclitaxel + Carboplatin	yes
3	62	Ovary	Serous carcinoma	High	IV	Pt-resistant relapse	weekly Paclitaxel + Bevacizumab	yes
4	63	Ovary	Serous carcinoma	High	III	Pt-sensitive relapse	Paclitaxel + Carboplatin	yes
5	57	Ovary	Serous carcinoma	High	III	Pt-resistant relapse	weekly Paclitaxel + Bevacizumab	yes
6	55	Endometrium	Endometrioid adenocarcinoma	Grade 3	IV	2nd line	weekly Paclitaxel	yes
7	47	Ovary	Clear cell carcinoma	High	III	Pt-resistant relapse	weekly Paclitaxel	yes
8	64	Endometrium	Serous carcinoma	High	III	1st tline	Paclitaxel + Carboplatin	yes
9	55	Ovary	Serous carcinoma	High	II	Pt-sensitive relapse	Paclitaxel + Carboplatin	yes
10	82	Endometrium	Endometrioid adenocarcinoma	Grade 3	III	Pt-sensitive relapse	Paclitaxel + Carboplatin	yes
11	57	Ovary	Serous carcinoma	High	III	adjuvant	Paclitaxel + Carboplatin	no
12	32	Ovary	Undifferentiated adenocarcinoma	High	IV	Pt-resistant relapse	weekly Paclitaxel	yes
13	52	Ovary	Endometrioid adenocarcinoma	Grade 2	I	Adjuvant	Paclitaxel + Carboplatin	no
14	77	Cervix	Epidermoid carcinoma	NS	IV	2nd line	weekly Paclitaxel	yes
15	45	Endometrium	Carcinosarcoma	High	I	1st line	Paclitaxel + Carboplatin	no
16	83	Ovary	Serous carcinoma	High	III	Neoadjuvant	Paclitaxel + Carboplatin	no
17	72	Ovary	Serous carcinoma	High	III	Pt-resistant relapse	weekly Paclitaxel	yes
18	71	Endometrium	Endometrioid adenocarcinoma	Grade 3	I	Adjuvant	Paclitaxel + Carboplatin	no
19	74	Ovary	Serous carcinoma	High	III	Pt-resistant relapse	weekly Paclitaxel	yes
20	61	Ovary	Clear cell carcinoma	High	I	Adjuvant	Paclitaxel + Carboplatin	no

Grade: Depends on the cancer aggressiveness—for endometrial and endometrioid-type ovarian tumors, grades are classified according to the classic system from 1 to 3. For ovarian tumors, the current high-grade versus low-grade grading system is shown. Stage: International Federation of Gynecology and Obstetrics (FIGO) classification scale from I to IV. Strategy: For endometrial and cervical tumors, it is reported whether it was for localized disease (adjuvant) or the number of chemotherapy lines for advanced disease (1st line or 2nd line). For ovarian tumors, it is reported if the strategy was for localized disease (adjuvant or neoadjuvant) or advanced disease according to previous platinum sensitivity (platinum-sensitive or platinum-resistant). Refractory cancers were categorized as platinum-resistant. Treatment: weekly paclitaxel (80 mg/m^2^) was administered at days 1, 8, and 15 every 21 days. Three weekly paclitaxel (175 mg/m^2^) + carboplatin (AUC5) was administered in day 1 every 21 days. Abbreviation: NS: not specified; Pt-sensitive: platinum-sensitive; Pt-resistant: platinum-resistant.

## Data Availability

The data presented in this study are available on request from the corresponding author.

## Supplementary Materials

The following supporting information can be downloaded at: https://www.mdpi.com/article/10.3390/cancers14051146/s1, Supplementary file: un-cropped images of the original western blots from which figures have been derived.
