# Supplementary material for: Paclitaxel Induces Epidermal Molecular Changes and Produces Subclinical Alterations in the Skin of Gynecological Cancer Patients
Source: Cancers (Basel). 2022 Feb 23;14(5):1146. doi: 10.3390/cancers14051146 (PMC8909563; doi:10.3390/cancers14051146)

un-cropped images of the original western blots from which figures have been derived

Figure S1

AQP3 **β -actin**

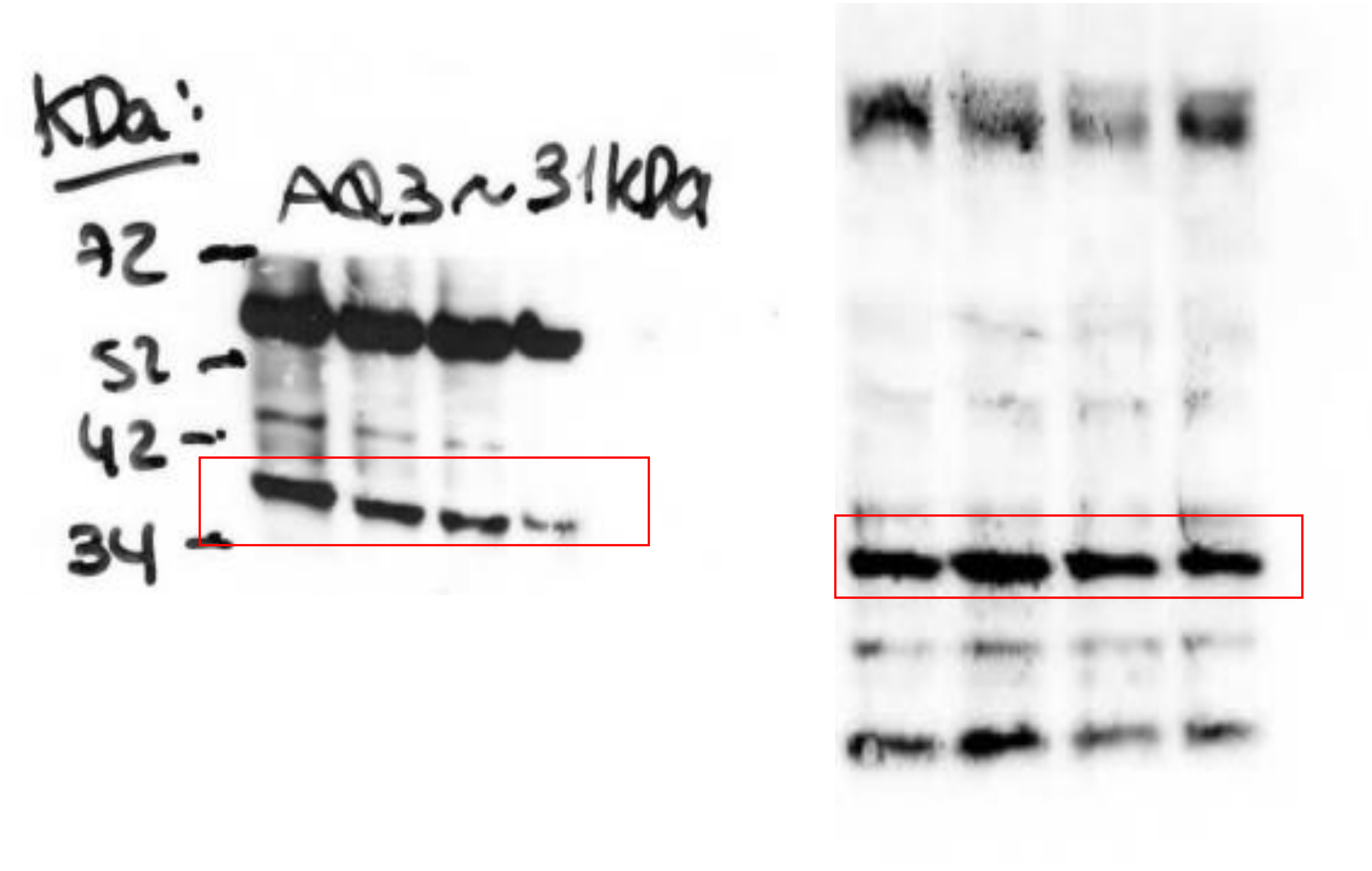

FIGURE 2

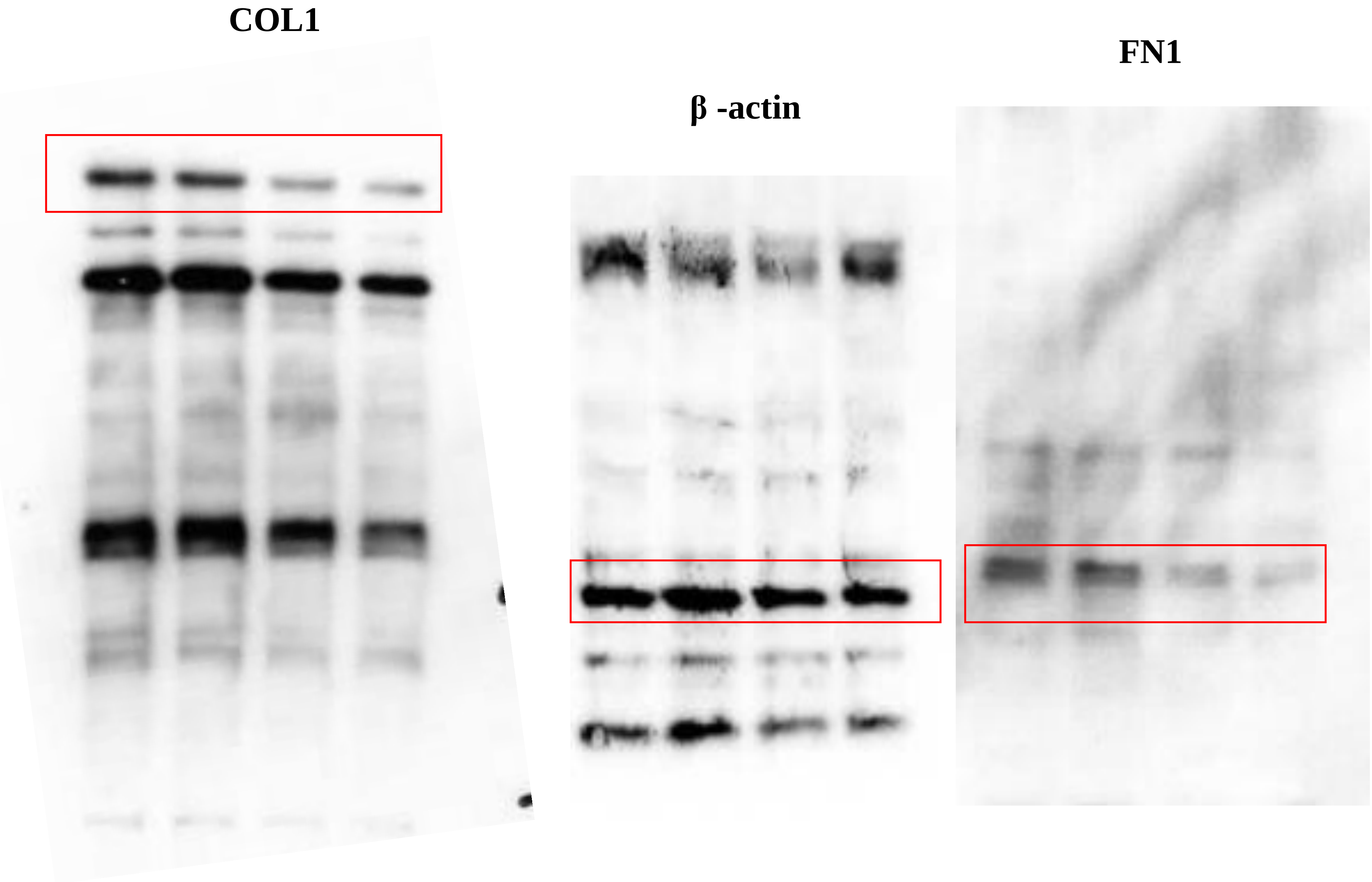

Figure S2

ELN

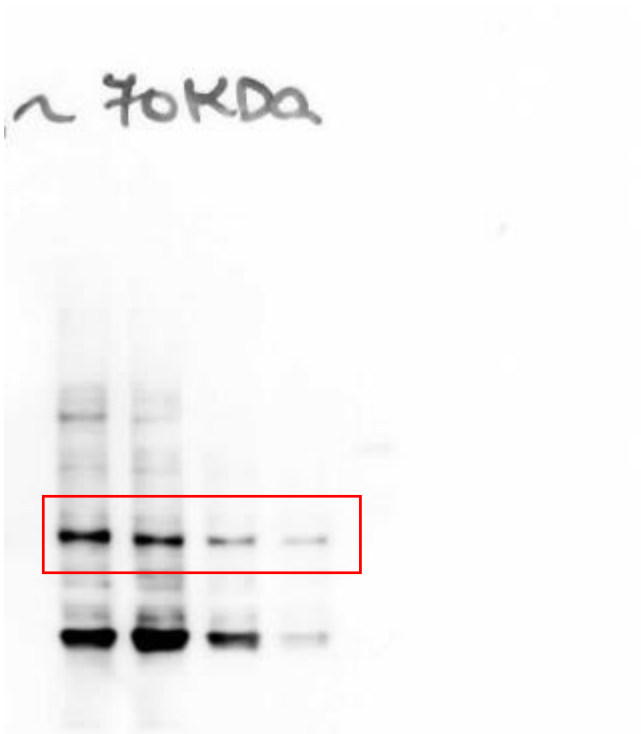

β-actin

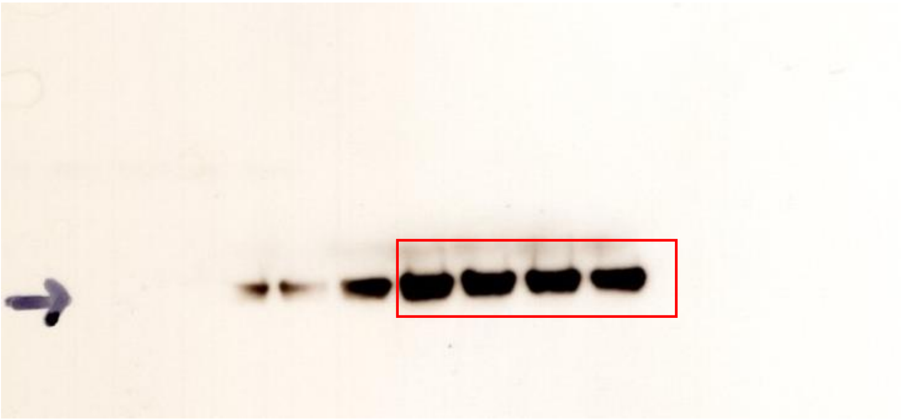

Supplement: Supplementary file 1 [file cancers-14-01146-s001.zip › cancers-1524863-supplementary.pdf]
